# Intracellular PRRs Activation in Targeting the Immune Response Against Fungal Infections

**DOI:** 10.3389/fcimb.2020.591970

**Published:** 2020-10-20

**Authors:** Grasielle Pereira Jannuzzi, José Roberto Fogaça de Almeida, Larissa Neves Monteiro Paulo, Sandro Rogério de Almeida, Karen Spadari Ferreira

**Affiliations:** ^1^Departamento de Análises Clínicas, Faculdade de Ciências Farmacêuticas da Universidade de São Paulo, São Paulo, Brazil; ^2^Departamento de Ciências Biológicas do Instituto de Ciências Ambientais, Químicas e Farmacêuticas, Universidade Federal de São Paulo, Diadema, Brazil

**Keywords:** fungal infection, intracellular receptors, PRRs, innate immune response, nucleic acids

## Abstract

The immune response against fungal infections is complex and exhibits several factors involving innate elements that participate in the interaction with the fungus. The innate immune system developed pattern recognition receptors that recognize different pathogen-associated molecular patterns present both on the surface of the fungi cell wall and on their genetic material. These receptors have the function of activating the innate immune response and regulating a subsequent adaptive immune response. Among pattern recognition receptors, the family of Toll-like receptors and C-type lectin receptors are the best described and characterized, they act directly in the recognition of pathogen-associated molecular patterns expressed on the wall of the fungus and consequently in directing the immune response. In recent years, the role of intracellular pattern recognition receptors (TLR3, TLR7, TLR8, and TLR9) has become increasingly important in the pathophysiology of some mycoses, as paracoccidioidomycosis, cryptococcosis, aspergillosis, and candidiasis. The recognition of nucleic acids performed by these receptors can be essential for the control of some fungal infections, as they can be harmful to others. Therefore, this review focuses on highlighting the role played by intracellular pattern recognition receptors both in controlling the infection and in the host's susceptibility against the main fungi of medical relevance.

## Introduction

Fungi are eukaryotic cells with composition predominantly of carbohydrate polymers interspersed with glycoproteins and complex morphogenesis. Some fungal species have the capacity to present different forms depending on the temperature in which they are, in other words, they exhibit thermal dimorphism, which can facilitate the evasion of the immune response and dissemination in the host.

Depending on the morphotype, conidia or yeast, growth stage and of the species, the fungus can express different molecular patterns associated with pathogens (PAMPs) on the surface, which will be recognized by the cells of the immune system (Bowman and Free, 2006; Levitz, 2010; Romani, 2011; Gow and Hube, 2012; Gow et al., 2012).

The major fungi of medical relevance exhibit a wall composed mainly of β-glucans, chitins and mannans. β-glucans are glucose polymers, where in the β-(1,3)-glucan form is considered to be its major fungal cell wall structure and has varying numbers of β-(1,6)-glucans, chitin is an *N*-acetylglucosamine polymer, and mannans is composed of chains with hundreds of mannose molecules that are added in the fungi proteins via *N* or *O*-linkages (Bowman and Free, 2006; Wheeler and Fink, 2006; Romani, 2011).

In addition to the fungal cell wall components, nucleic acids (NAs) are also considered to be true PAMPs capable of inducing strong stimuli to initiate a potent immune response (Bacci et al., 2002; Yordanov et al., 2005; Ramirez-Ortiz et al., 2008; Eberle et al., 2009; Freund et al., 2019). Pathogen derived NAs are recognized differently from self NAs, leading into account some types of parameters, such as location, sequence, structure, and molecular modifications. On the other hand, self NAs such as extranuclear DNA or extracellular RNA can be recognized as DAMPs because they are reliable indicators of cell damage (Pichlmair and Reis e Sousa, 2007; Chen and Nunez, 2010; Takeuchi and Akira, 2010; Barbalat et al., 2011).

The innate immune response uses its mechanisms quickly and conserved in response to a wide variety of fungal pathogens. Thus, the innate immune system developed receptors called pattern recognition receptors (PRRs), which are responsible for recognizing both PAMPs located on the surface of pathogens, and NAs that are located intracellularly.

The most well-characterized PRRs involved in sensing and recognition of fungal comprise 5 families, toll-like receptors (TLRs), C-type lectin-like receptors (CLRs), NOD-like receptors (NLR), galectins family proteins (Galectin-3), and scavenger receptors (such as CD5 and CD36) (Yoneyama et al., 2004; van de Veerdonk et al., 2008; Jouault et al., 2009; Bourgeois et al., 2010; Romani, 2011; Plato et al., 2015). Each of these receptors recognizes different PAMPs present on the fungal cell wall surface as well as its genetic material (Table 1).

**Table 1:** List of PRRs, their ligands, and respective fungi.

**Receptor**	**Ligand**	**Most fungal species**
TLR1/2	Triacylated lipoprotein	*A. fumigatus, C. albicans*
	GXM	*C. neoformans*
TLR2	Phospholipomannan	*C. albicans*
	β-1,2-oligomannoside	*C. albicans, S. schenkii, S. brasiliensis, P. brasiliensis*
TLR2/6	Diacylated lipoprotein	*A. fumigatus, C. albicans*
	GXM	*C. neoformans*
TLR3	dsRNA	*A. fumigatus, P. brasiliensis, C. neoformans*
TLR4	*O*-linked mannosyl, mannan	*C. albicans, S. serevisiae, S. brasiliensis, P. brasiliensis*
	GXM	*C. neoformans*
TLR7	ssRNA	*C. albicans*
TLR8	ssRNA	*C. albicans*
TLR9	DNA	*A. fumigatus, C. neoformans, Candida, P. brasiliensis, M. furfur*
Dectin-1	β-1,3-glucans	*A. fumigatus, C. albicans, P. carinii, S. schenckii, F. pedrosoi, C. neoformans, P. brasiliensis, H. capsulatum, Malassezia spp, S. cerevisiae, C. posadasii, T. mentagrophytes, F. solani, C. cladosporioides*
Dectin-2	High-mannose structures α-mannans	*C. albicans, C. glabrata C. neoformans, T. rubrum, Malassezia spp, H. capsulatum, F. pedrosoi, A. fumigatus* and *M. audouini*
Dectin-3	GXM	*C. neoformans serotype AD (C.n-AD)* and *C. gattii serotype B*
	α-mannans	*C. albicans*
Mannose receptor	α-glucans	*P. brasiliensis, C. yeast, C. neoformans Histoplama capsulatum, Blastomyces dermatitidis*
	Chitin	*Candida yeast, C. neoformans*
	Mananas	*A. fumigatus, S. cerevisiae, P. carinii, C. neoformans, P. brasiliensis, C. albicans, C. parapsilosis*, and *S. schenckii*
DC-SIGN	*N*-linked mannans	*Dermatophytes, A. fumigatus, S. cerevisiae, C. neoformans, C. albicans*, and *C. topicum*
	Galactomannans	*A. fumigatus*
MINCLE	a-mannose, glyceroglycolipid	*A. fumigatus, Malassezia spp., F. pedrosoi, P. carinii*, and *C. albicans*
	mannosyl fatty acids	*Malassezia spp P. carinii*
	MSG/gpA	*P. carinii*
Langerin	β-1,3-glucans	*M. furfur, S. cerevisia, C. albicans, C. glabrata, C. krusei, C. parapsilosis*, and *C. tropicalis*
Galectin-3	a-mannosides	*C. albicans*

*GXM, Glucuronoxylomannan; dsRNA, Double-stranded RNA; ssRNA, Single-stranded RNA; MSG/gpA, glycoprotein of P. carinii*.

The PRRs differ in signal transduction after the recognition of the fungal antigen and in its subcellular location. After the recognition of their respectives ligands, these receptors initiate the activation of the innate and adaptive immune response in order to induce a protective response against the fungus (Perruccio et al., 2005; Plato et al., 2015), but this is not always possible.

Among the PRRs, intracellular TLRs play an important role in the recognition of NAs from fungi and has been showing a potential activator of the immune response, which can mediate a protective response for the host or favor the escape of the fungus, with consequent dissemination and worsening of the disease (Carvalho et al., 2012; Menino et al., 2013; Jannuzzi et al., 2019). In addition to the pathophysiological context, the activation of intracellular TLR has been used as a promising technique for the treatment of some fungal infections (de Sousa et al., 2014; Morais et al., 2016; Freund et al., 2019). Thus, in this review, we will discuss the role of receptors involved in the recognition of fungal NAs, their location, signaling and the role played in the host's immune response.

## Endosomal TLRs Involved in the Recognition of Fungal NAs

The presence of TLRs in host defense has been described for several fungal pathogens such as *Candida albicans* (*C. albicans*) (Netea et al., 2002, 2006), *Aspergillus fumigatus* (*A. fumigatus*) (Meier et al., 2003; Dubourdeau et al., 2006), *Cryptococcus neoformans* (*C. neoformans*) (Yauch et al., 2004; Biondo et al., 2005), *Fonsecaea pedrosoi* (*F. pedrosoi*) (Sousa et al., 2011) and *Paracoccidioides brasiliensis* (*P. brasiliensis*) (Ferreira et al., 2007; Loures et al., 2015; Jannuzzi et al., 2019). These receptors act as a molecular button to trigger the activation of innate immunity and regulate a subsequent adaptive immune response, essential in the control of infections (Kawai and Akira, 2011).

TLRs are a family of receptors that comprise up to now 12 functional proteins identified in mice and 10 in humans, TLR1-9/TLR11-13, and TLR1-10, respectively (Akira et al., 2006; Medzhitov, 2007), of which TLR3, TLR7, TLR8, and TLR9 are found intracellularly, and recognize NAs derived from pathogens uptake by endocytosis or derivatives of autophagy and transferred to the endolysosomal compartment (Blasius and Beutler, 2010; Barbalat et al., 2011; Lee et al., 2012; Schuberth-Wagner et al., 2015).

**Figure 1:**
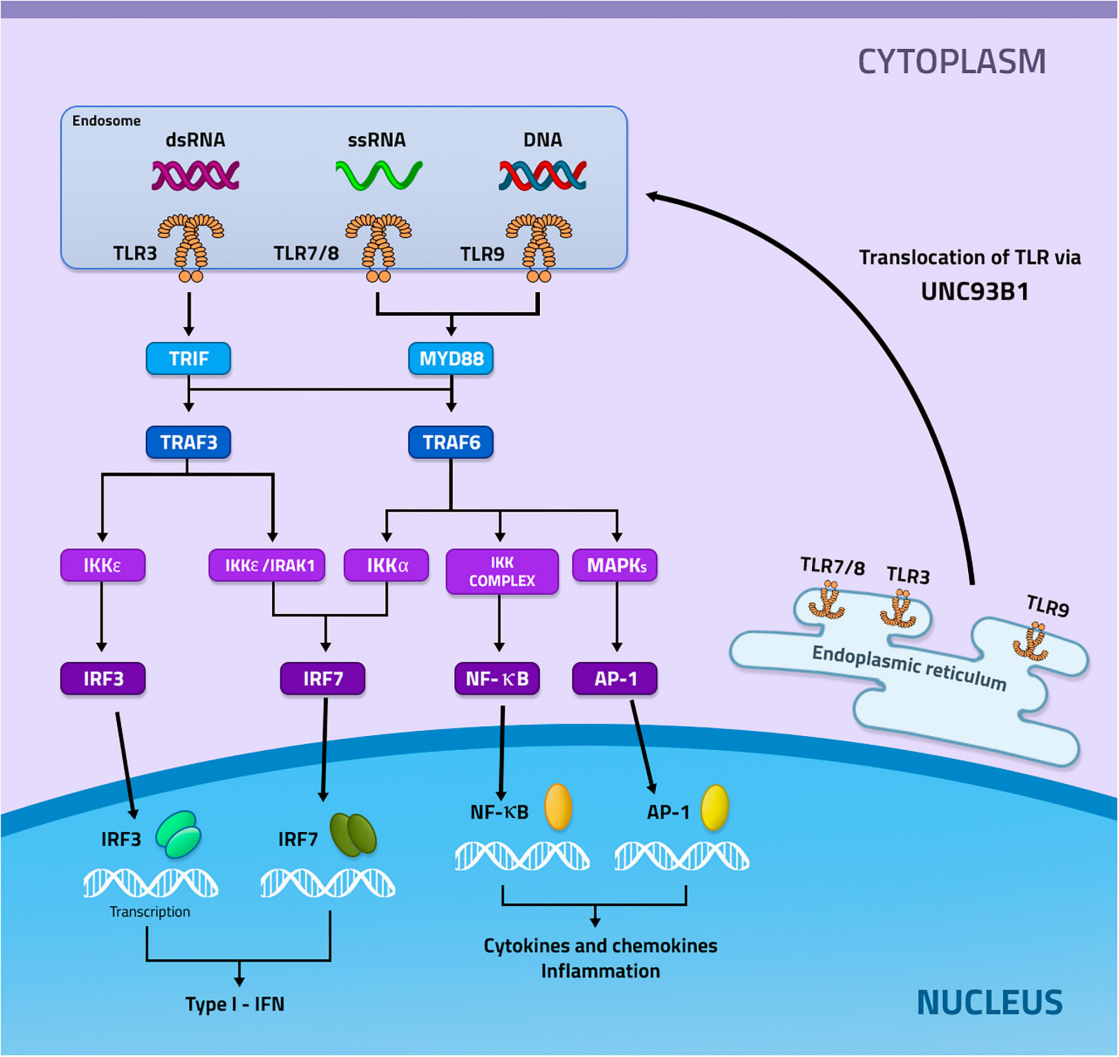
Signaling mediated by the activation of TLR3, 7 and 9. TLRs 3, 7, and 9 are recruited from the endoplasmic reticulum through the UNC93b1 protein to the endosome. In the endosome these receptors will recognize their specific ligands, in which TLR3 recognizes dsRNA, while TLR7 ssRNA, and TLR9 DNA. Then each of these receivers, now activated, will start different signaling pathways. TLR3 recruits TRIF with subsequent activation of TRAF3. TRAF3 can activate two ways, the first IKKε will be activated and induce the activation of IRF3, while the second will occur activation of IKKε/IRAK1 which will result in the activation of IRF7. TLR7 and TLR9 recruit MYD88 which will result in the activation of TRAF6. TRAF6 may induce 3 different signals, the first will occur the activation of IRF7 through Ikka, the second will occur the activation of the IKKs complex that will induce the activation of NF-κB and the last will occur the activation of MAPKs that will mediate the activation of AP−1. IRF3 and IF7 will be transcribed in the nucleus inducing the production of type I IFN, whereas NF- κB and AP-1 will be transcribed and will mediate the production of inflammatory cytokines and chemokines.

Endosomal TLRs are synthesized in the endoplasmic reticulum and subsequently transported to Endosome or Lysosome with the help of the uncoordinated 93 homolog B1 (UNC93B1), which in addition to performing the transport it also cooperates with the expression and stabilization of the endosomal TLRs (Lee and Barton, 2014; Pelka et al., 2018). TLRs can be located in both endosomes and lysosomes, performing monitoring of endolysosomal contents or detecting NAs in the cytoplasm (Kawai and Akira, 2006; Barbalat et al., 2011) (Figure 1).

Intracellular TLRs recognize the different types of NAs liberated from fungi within the phagosome can stimulate or modulate the host response during infection. TLR3 recognizes double-stranded RNA (dsRNA) without requiring specific sequences, however, a minimum length of 40 base pairs is required for binding and hence induction of TLR3 responsiveness. TLR3 is activated by dsRNA from *A. fumigatus* conidia, yeast *C. albicans*, yeast *P. brasiliensis*, among others which will be discussed later (Bourgeois et al., 2011; Beisswenger et al., 2012; Carvalho et al., 2012; Jannuzzi et al., 2019). This receptor also recognizes polyinosine-polycytidylic acid [poly (I: C)] synthetic synthetic RNA (Alexopoulou et al., 2001; Liu et al., 2008).

Both TLR7 and TLR8 recognize single-stranded RNA (ssRNA), however this recognition exhibits distinction sequences motifs in ssRNA. While TLR7 recognizes GU-rich sequences (e.g., UUGU, GUUC), TLR8 requires AU-rich sequences (e.g., AUUU, UAUC) (Vollmer et al., 2005; Forsbach et al., 2008). TLR7 is involved in the recognition of ssRNA from *Candida spp* and *F. pedrosoi* (Bourgeois et al., 2011; Sousa et al., 2011).

TLR9 recognizes nomethylated cytosine-guanosine (CpG) motifs in DNA (Hemmi et al., 2000). CpG motifs were first described in bacterial DNA, evidencing their immunostimulatory capacity, however, it is now known that the recognition of these motifs is also related to many viruses and fungi (Hemmi et al., 2000; Souza et al., 2001; Barton, 2007). Among the studied fungal infections, TLR9 activation was related to DNA from *A. fumigattus, C. albicans, P. brasiliensis, S. cerevise, M. furfur*, and *C. neoformans* (Nakamura et al., 2008; Ramirez-Ortiz et al., 2008; Kasperkovitz et al., 2010, 2011; Menino et al., 2013).

## Endosomal TLR-Mediated Signaling

After the recognition of NAs, a signaling cascade begins that will produce pro-inflammatory cytokines that are important for the recruitment and activation of immune cells (Kawai and Akira, 2010).

The recognition of endosomal TLRs by their ligands will induce the recruitment of different adapter proteins at the beginning of signaling. TLR7, 8, and 9 will recruit the adapter protein Myeloid differentiation primary response 88 (MYD88), which will activate the TNF receptor associated factor 6 (TRAF6) protein. TRAF6 can activate 3 distinct pathways, in which activating kappa-B kinase subunit alpha (IKKα) will induce the activation of interferon regulatory factor 7 (IRF7), which will be translocated to the nucleus inducing the production of Type I Interferons (type I IFN). If TRAF6 activates the IKKs it will generate the activation of the nuclear factor κB (NF-kB), which will be transcribed in the nucleus and induce the production of pro-inflammatory cytokines and chemokines. However, if TRAF6 activates mitogen-activated protein kinases (MAPKs), transcription of activator protein 1 (AP1) will occur, which will also result in the production of pro-inflammatory cytokines and chemokines. TLR3, on the other hand, recruits the adapter protein TIR-domain-containing adapter-inducing interferon-β (TRIF), which will activate the TRAF3 protein that can activate 2 different pathways, one that will have IKKε/IRAK activation and subsequent activation of IRF7, which will be transcribed in the nucleus and culminate in the production of type I IFN. The other pathway will have IKKε/TBK1 activation which will induce the activation of IRF3, which will be transcribed in the nucleus and will also result in the production of type I IFN (Figure 1) (Fitzgerald et al., 2003; Meylan et al., 2004; Conze et al., 2008; Kawai and Akira, 2010; Lee et al., 2012; Yamashita et al., 2012; Gay et al., 2014).

## Expression of Intracellular TLRs in Cells of the Immune System

Intracellular TLRs are expressed mainly in phagocytes cells, TLR3 is expressed in myeloid dendritic cells, macrophages, epithelial cells, endothelial cells, and fibroblasts. Among the DC subtypes, TLR3 is highly expressed in conventional DCs (CD8α^+^) from mice and human DCs (CD141^+^), while plasmacytoid DCs express TLR7 and TLR9, but not TLR3 (Matsumoto and Seya, 2008; Beisswenger et al., 2012; Carvalho et al., 2012; Jannuzzi et al., 2019). Both TLR7 and TLR9 are expressed in monocytes, macrophages, DCs, and B lymphocytes (Ramirez-Ortiz et al., 2008; Mancuso et al., 2009; Biondo et al., 2011, 2012; Bourgeois et al., 2011; Kasperkovitz et al., 2011). TLR9 is also activated in neutrophils (Bellocchio et al., 2004), while TLR8 is expressed in all myeloid cells (Bellocchio et al., 2004; Ganguly et al., 2009).

## Participation of Intracellular TLRs in Fungal Infections

In this section, we will discuss the role of TLR3, TLR7, and TLR9 in the main fungal infections, such as candidiasis, aspergillosis, paracoccidioidomycosis, cryptococcosis, and histoplasmosis.

## TLR3

TLR3 detects endogenous dsRNA released by cells in the death process from necrosis (Cavassani et al., 2008). Thus, it is possible that TLR3 is activated when observing damage in the host caused by an infectious process, and after its activation, it plays a role in regulating inflammatory, adaptive memory and tolerance response (Carvalho et al., 2012).

In aspergillosis, the TLR3 role in DCs is of great relevance, being critical both for its maturation and the production of type I IFN. TLR3 in both murine and human DCs by recognizing the fungus RNA effectively induces the CD8 T cell primer for an MHC-I restricted protective memory response against the fungus. The absence of TLR3 directly impacts the migration of murine DCs from the lung to the lymph nodes, due to the failure in the expression of *CCR7*, which leads to a deficient activation of T cells. In mice, the susceptibility to aspergillosis is increased in conditions of TLR3 absence, leading to an increase in the inflammatory process in murine aspergillosis, accompanied by a decrease in the production of IFN-γ, IL-10. In humans, the single nucleotide polymorphism (SNP) of TLR3 provides more invasive aspergillosis. Patients carrying a TLR3 +95C/A exhibit a phenotype of loss of function of DCs, which is correlated with a severe infection by *A. fumigatus* and deficiency in the activation of CD8 T cells (Carvalho et al., 2012). The therapeutic efficacy with micafungin in aspergillosis is strictly induced by the activation of the TLR2/dectin-1 and TLR3/TRIF signaling pathways, which regulate the inflammatory/anti-inflammatory balance during infection, mainly by increasing the production of IL-10 and decreased TNF-α (Moretti et al., 2014).

Human endothelial cells (ECs) express several genes after their interaction with *C. albicans* yeasts, such as genes involved in cell migration, proliferation, among others. TLR3 is involved as a mediator in the expression of the CXCL8/18 gene by ECs, such gene acts in the protective pro-inflammatory endothelial response in the candidiasis (Müller et al., 2007). Patients with the L412F genetic variant of TLR3 are more susceptible to chronic mucocutaneous candidiasis (CMCC). Human peripheral blood mononuclear cells (PBMCs) that carry L412F after interaction with *C. albicans* have a reduction in the production of TNF-α, type I IFN and IFN-γ (Nahum et al., 2012), these cytokines are extremely important for maintaining the innate and adaptive response against candidiasis (Gozalbo and Gil, 2009), this low production can strengthen the susceptibility of part of the patients with CMCC to infection with *C. albicans* (Nahum et al., 2012).

Contrary to other fungal infections, in paracoccidioidomycosis (PCM) TLR3 is used as an escape mechanism by *P*. *brasiliensis*, generating greater susceptibility to the disease. Although TLR3 does not play a role in the phagocytosis of *P brasiliensis* by murine BMDMs (Jannuzzi et al., 2019), as observed in the phagocytosis of *C. neoformans* by microglia cells (Redlich et al., 2013), in the absence of TLR3 BMDMs have greater microbicidal activity with increased NO and decreased fungal burden. In murine PCM, the absence of TLR3 generated greater resistance to infection, with decreased pulmonary fungal burden and an increased protective response mediated by IFN-γ and IL-17-producing CD8T cells (Jannuzzi et al., 2019). CD8 T lymphocytes, as well as IFN-γ, and IL-17 cytokines, are involved with the protective response of PCM (Loures et al., 2009, 2010, 2014; Jannuzzi et al., 2015). The role of TLR3 in each infection discussed in that session is summarized in Table 2.

**Table 2:** Response induced by TLR3 activation.

**Disease**	**Effect**	**Outcome**
Aspergillosis	Expression CCR7 in BMDC CD8 T cell primer for an MHC-I restricted protective memory response	Protective response
TLR3 + 95C/A SNP Invasive Aspergillosis	Improved phenotype and functions in Human DC CD8 T cell primer	Protective response
Candidiasis	Expression CXCL8/18 gene in Human EC	Protective response
L412F–TLR3 SNP Chronic mucocutaneous candidiase	Increase production of TNF-α, type I IFN and IFN-γ by Human PBMCs	Protective response
Cryptococcosis	No effect in Microglia	–
Paracoccidioidomycosis	Decrease both microbicidal activity, NO production and increase fungal burden by BMDM Decrease IFN-γ and IL-17-producing CD8T cells	Susceptibility

*BMDC, bone marrow-derived dendritic cells; DC, dendritic cells; EC, endothelial cells; PBMCs, peripheral blood mononuclear cells; BMDM, bone marrow-derived macrophage*.

## TLR7

The involvement of TLR7 in ssRNA recognition and its role in the host's defenses against viruses and bacteria (Diebold et al., 2004; Mancuso et al., 2009) is well-known, it has recently been demonstrated that this receptor also plays an important role in the recognition and targeting of the immune response against some pathogens fungal (Biondo et al., 2012).

The recognition of *Candida* spp RNA by TLR7 proved to be crucial in inducing the type I IFN response in bone marrow-derived dendritic cells (BMDCs) (Bourgeois et al., 2011). The activation of the type I IFN response is essential for the maturation of DCs and participates in the polarization of the adaptive immune response, inducing the differentiation of Th cells (Stetson and Medzhitov, 2006). The release of high levels of IFN-β by BMDCs challenged with *Candida* spp depends on TLR7/MYD88/IRF1 and Src/Syk-mediated signaling. However, in a disseminated candidiasis model, type I IFN signaling promotes the persistence of *C. glabrata* in the host, being detrimental to the fungus clearance (Bourgeois et al., 2011). In a model of *C. albicans* infection, resistance to infection it was also correlated with the activation of TLR7 and the transcription factor IRF1 (Bacci et al., 2002; Biondo et al., 2012).

In studies with *Histoplasma capsulatum* (*H. capsulatum*) it has already been shown that macrophages and dendritic cells play different roles in control and infection evolution. While the fungus survives and replicates within the macrophages, in DCs its growth is restricted (Gildea et al., 2001). This fact may be related to the capacity of these cells to produce type I IFN since it was observed that macrophages stimulated with *H. capsulatum* do not produce this type of cytokine while DCs do it. The type I IFN mediated response is necessary for DCs to be able to restrict the growth of intracellular fungi and survive the fungal infection. Type I IFN production by BMDCs is directed by both TLR7 and TLR9. TLR7-deficient BMDCs exhibit a significant decrease of type I IFN when stimulated with yeast from *H. capsulatum*, the same could be observed with TLR9-deficient BMDCs, however, BMDCs deficient in both TLR7/TLR9 the levels of type I IFN are significantly reduced when compared to the deficient ones separately, this decrease is accompanied by increased fungal growth in the cell and cell lysis. In addition, these TLR7/TLR9 deficient BMDCs failed to induce activation of CD4^+^ T cells. In the histoplasmosis model, TLR7/TLR9-deficient animals show a more aggressive infection, exhibiting increased neutrophil recruitment, increased lung damage, colonization in the brain, and consequently increased death (Van Prooyen et al., 2016).

The mechanisms present in the susceptibility of chromoblastommycosis are involved with a defect in the recognition of the pathogen by the innate immune response. The *F. pedrosoi* is recognized by CLRs, but there is a failure in the co-stimulation of TLRs leading to chronic infection. Thus, in an attempt to reverse this profile, in 2011, our group showed that animals infected with *F. pedrosoi* after being treated with topical administration of imiquimod, a TLR7 agonist, had a reduction in the fungal load on the skin, suggesting that the activation of TLR7 would be important in protecting the disease. In 2014, the administration of imiquimod was used in four patients with chromoblastomycosis who had a decrease in the diameter of the lesions and a clearing of the pathogen, confirming our previous hypothesis (de Sousa et al., 2014). The role of TLR7 in each infection discussed in that session is summarized in Table 3.

**Table 3:** Response induced by TLR7 activation.

**Disease**	**Effect**	**Outcome**
Candidiasis (*C. albicans*)	Type I IFN production BMDCs	Protective response
Histoplasmosis	Improved Type I IFN production BMDCs	Protective response
Chromoblastommycosis	Reduction in the fungal load on the skin Decrease in the diameter of the lesions	Protective response

*BMDC, bone marrow-derived dendritic cells*.

## TLR9

The ability to induce phagosomal recruitment of TLR9 is conserved in distinct fungal taxonomic groups after phagocytosis, such as *A. fumigattus, C. albicans, S. cerevise, M. furfur*, and *C. neoformans* (Kasperkovitz et al., 2010, 2011). It has recently been shown that TLR9 recruitment and accumulation in the *A. fumigattus* and *C. albicans* phagosome is dependent on the recognition of β1,3-glucan by dectin-1/syk pathway. This pathway mediates phagosome acidification and allows recruitment and retention of TLR9 in this compartment. Knowing that TLR9 needs cysteine, L cathepsin and S cathepsin lysosomal proteases for their phagosome cleavage, it has been seen that blocking Dectin-1/syk-dependent phagosomal acidification therefore blocks the activation of lysosomal cathepsins, which will inhibit TLR9 cleavage. Thus, TLR9 can be recruited for the β1,3-glucan-containing phagosome, but if there is impairment in phagosome acidification there will be no cleavage it will not be activated. Thus, TLR9 modulates gene expression in a Dectin-1 dependent form in response to β1,3-glucan (Khan et al., 2016).

The DNA of *C. albicans*, when internalized, is located inside the endosomal and lysosomal compartments together with the CpG oligodeoxynucleotides (CpG-ODN). This DNA is capable of activating BMDCs through TLR9/MyD88-mediated signaling, but using a mechanism independent of the unmethylated CpG motif. The activation of BMDCs, by the DNA of *C. albicans*, induces immunostimulatory effects, such as IL-12p40 production, CD40 expression and NF-κB activation (Kasperkovitz et al., 2011). The same effects are also seen with the DNA of *C. neoformans* and *A. fumigatus* in BMDCs (Nakamura et al., 2008; Ramirez-Ortiz et al., 2008). In bone marrow-derived macrophages (BMDM), TLR9-mediated signaling with *C. albicans* DNA does not have an effect on their antifungal effector functions. However, the absence of TLR9 improves the effective antifungal response of both BMDM and PBMC, inducing an increased production of TNF-α, IL-6, and nitric oxide and decreasing the production of IL- 10, in addition to improving the microbicidal activity of BMDMs. Interestingly, the systemic infection by *C. albicans* in TLR9^−/−^ mice did not show any difference in the fungi load of the analyzed organs or in their survival, when compared to the control group (van de Veerdonk et al., 2008; Miyazato et al., 2009). In contrast, TLR9 deficiency significantly increased resistance to mucosal candidiasis and reduced the growth of the fungal load on the analyzed organs (Bellocchio et al., 2004). The involvement of TLR9 during systemic infection by *C. albicans* yeasts may not impact the host's defense mechanism, being only related to the regulation of the immune response. The recognition of *C. albicans* suggests that multiple interactions between PAMPs and PRRs are integrated, acting synergistically and antagonistically, thus allowing the immune system to respond to this pathogen in a more specific way (Trinchieri and Sher, 2007; Netea et al., 2008; Miyazato et al., 2009).

The synthetic oligodeoxynucleotides (ODNs) containing CpG-rich motifs found in *A. fumigatus* DNA are capable of stimulating TLR9 influencing the host response to the fungal challenge. It was demonstrated that DNA obtained from *A. fumigatus* stimulates potently TLR9-dependent responses in BMDCs and human plasmacytoid dendritic cells, showing high production of proinflammatory cytokines like TNF-α and IL-12. The absence of TLR9 in BMDCs it abolished the production of these cytokines when stimulated with DNA from *A. fumigatus*, proving the role of TLR9 in the effector functions mediated by these cells (Ramirez-Ortiz et al., 2008). However, the activation of TLR9 in PMN does not play a role in the response against the fungus. Stimulation with CpG-ODN of *A. fumigatus* does not alter the antifungal effector functions in PMNs, the intracellular production of reactive oxygen intermediates (ROI), and the degranulation of these cells remaining unchanged. However, the absence of TLR9 in PMNs shows an increase in both conidiocidal activity and in hyphal damage activity, with increased azurophil granules degranulation (Bellocchio et al., 2004). In addition, TLR9^−/−^ mice exhibit less pulmonary fungal load, accompanied by a milder pulmonary inflammatory process, in addition to having better survival compared to control animals. Suggesting that, although TLR9 activation plays a different role, depending on the cell analyzed, its absence generates a protective response against aspergillosis (Bellocchio et al., 2004; Ramirez-Ortiz et al., 2008). In humans, susceptibility to allergic bronchopulmonary aspergillosis has been associated with a SNP in the C allele at T-1237C, located within the putative promoter of the TLR9 gene (Carvalho et al., 2008).

The purified DNA of *P. brasiliensis* activates TLR9 in macrophages, leading to the expression of cytokines and promoting their phagocytic capacity, while stimulation of macrophages with *P. brasiliensis* yeasts exhibits low TLR9 activation. The absence of TLR9 in macrophages leads to a decrease in the phagocytic capacity of yeasts. This suggests under physiological conditions, TLR9 can recognize the DNA of *P. brasiliensis* released from the dead fungus in the extracellular environment or after the live cells of the fungus are phagocyted by the immune cells. This entire process would result in the activation of TLR9 and could contribute to targeting the host's defense response against *P. brasiliensis*. In the context of infection by *P. brasiliensis*, the absence of TLR9 increases the susceptibility of mice at the beginning of the infection (48 h), generating an exacerbated inflammatory response with the increased neutrophil influx and high levels of TNF-α at the site of infection (Menino et al., 2013). This condition is harmful to the host due to the excessive release of oxidants, proteases and the intense increase in neutrophils, which can be responsible for organ damage and fungal sepsis (Bellocchio et al., 2004; Zelante et al., 2007).

The use of CPG as an adjuvant in vaccines is a strategy widely used against some models of infection, as it has great potential to induce and increase Th1 type immune response through TLR9 activation (Klinman et al., 2004; Latz et al., 2004; Krieg, 2006). In paracoccidioidomycosis, the combination of CPG and rPb27, a recombinant protein from *P. brasiliensis*, has shown promise in protection in the early stages of the disease (30 DPI), with a 98% reduction in fungal burden. The activation of TLR9 with the adjuvant CPG directed an intense Th1 immune response, with increased recruitment of lymphocytes, and the production of pro-inflammatory cytokines. Regarding macrophages, CPG increased the phagocytic response and microbicidal activity, as well as induced the production of IL-1β, TNF-α, IL-6 and IL-12, however *in vivo* the adjuvant decreased its recruitment, possibly due to increased efficiency of these stimulated cells (Morais et al., 2016). The role of TLR9 in each infection discussed in that session is summarized in Table 4.

**Table 4:** Response induced by TLR9 activation.

**Disease**	**Effect**	**Outcome**
Systemic candidiasis	IL-12p40 production, CD40 expression and NF- κB activation by BMDCs No effect in BMDM on their antifungal effector functions	No effect
Aspergillosis	Increase of TNF-α and IL-12 cytokine production by both BMDCs and human plasmacytoid dendritic cells No effect in PMN	Susceptibility
Paracoccidioidomycosis	Expression of cytokines and promoting phagocytic capacity in BMDM The combination of CPG and rPb27 induces 98% reduction in fungal burden	Protective response

*BMDC, bone marrow-derived dendritic cells; PMN, polymorphonuclear; BMDM, bone marrow-derived macrophage; CPG, nomethylated cytosine-guanosine; rPb27, recombinant protein from P. brasiliensis*.

## Conclusions

For years, the role of intracellular TLRs has been extensively studied in viral and bacterial infections (Diebold et al., 2004; Mancuso et al., 2009). However, these receptors have also been suggested for recognition and induction of immune responses against clinically relevant fungal pathogens (Bourgeois et al., 2010; Carvalho et al., 2012; Menino et al., 2013; Jannuzzi et al., 2019).

Intracellular TLRs are expressed mainly in phagocytic cells, such as macrophages and dendritic cells. These receptors are not necessary for the primary stage of detection and uptake of fungal pathogens (Bellocchio et al., 2004; Ramirez-Ortiz et al., 2008; Carvalho et al., 2012). However, after the recognition process on the surface of the phagocytic cells, the intracellular TLRs are recruited into phagosomes, and there perform the recognition of the NAs. The recognition of NAs, DNAs and RNAs, plays an important role in directing the murine immune response, both in innate and adaptive immunity. The type of induced response is particular for each fungus species and, depending on the recognized NAs, there will be the activation of a specific intracellular PRR that could culminate in a protective response or serve as an evasion mechanism of the immune system or, even, simply not influencing in the course of the disease (Bourgeois et al., 2010; Carvalho et al., 2012; Menino et al., 2013; Jannuzzi et al., 2019). Interestingly in humans, some SNRs in intracellular TLR are associated with greater susceptibility to fungal diseases in some types of immunocompromised patients (Carvalho et al., 2008, 2012). In addition the involvement in the immune response against fungal infections, the activation of these receptors is the target of therapeutic strategies, showing efficiency in the polarization of the protective response and resolution of signs and symptoms (de Sousa et al., 2014; Morais et al., 2016).

Based on the studies discussed in this review, we understand that although the signaling mediated by intracellular PRRs in fungal infections is not yet fully understood, the studies developed to date point to an important role in the activation of these TLRs. Thus, we suggest it is necessary to carry out further studies involving this type of signaling, which can help to better clarify the pathophysiology of fungal infections, in addition to contributing to the development of new therapeutic and prophylactic strategies.

## Author Contributions

The study was planned by KF, wrote by GJ and JA. The tables and figures were prepared by GJ, JA, and LP. The authors SA and KF discussed the session and the content covered. All authors reviewed the manuscript.

## Conflict of Interest

The authors declare that the research was conducted in the absence of any commercial or financial relationships that could be construed as a potential conflict of interest. The handling editor declared a shared affiliation with several of the authors GJ, JA, LP, and KF at the time of review.
